# Members of the Regulatory Lymphocyte Club in Common Variable Immunodeficiency

**DOI:** 10.3389/fimmu.2022.864307

**Published:** 2022-05-20

**Authors:** Sudhir Gupta, Yesim Demirdag, Ankmalika Abha Gupta

**Affiliations:** Basic and Clinical Immunology, Change affiliation of Ankmalika Abha Gupta to Division of Basic and Clinical Immunology, Irvine, CA, United States

**Keywords:** Treg, CD8 Treg, Breg, T follicular regulatory cells, CVID, autoimmunity, germinal center

## Abstract

The role of CD4 T regulatory cells is well established in peripheral tolerance and the pathogenesis of the murine model and human autoimmune diseases. CD4 T regulatory cells (CD4 Tregs) have been investigated in common variable immunodeficiency (CVID). Recently, additional members have been added to the club of regulatory lymphocytes. These include CD8 T regulatory (CD8 Tregs), B regulatory (Bregs), and T follicular helper regulatory (T_FR_) cells. There are accumulating data to suggest their roles in both human and experimental models of autoimmune disease. Their phenotypic characterization and mechanisms of immunoregulation are evolving. Patients with CVID may present or are associated with an increased frequency of autoimmunity and autoimmune diseases. In this review, we have primarily focused on the characteristics of CD4 Tregs and new players of the regulatory club and their changes in patients with CVID in relation to autoimmunity and emphasized the complexity of interplay among various regulatory lymphocytes. We suggest future careful investigations of phenotypic and functional regulatory lymphocytes in a large cohort of phenotypic and genotypically defined CVID patients to define their role in the pathogenesis of CVID and autoimmunity associated with CVID.

## Introduction

The major function of the immune system is to protect from foreign pathogens, allergens, and intrinsic aberrant malignant cells and maintain tolerance to self-antigens (1). Immune tolerance is maintained by central and peripheral tolerance (2, 3). Central tolerance occurs in the primary lymphoid organs (thymus and bone marrow), where T- or B-cell clones that recognize autoantigens with high affinity are deleted predominantly by apoptosis and by receptor editing in B cells. Peripheral tolerance that occurs in the secondary lymphoid organs (spleen, lymph nodes) involved suppression of effector functions of autoantigen-recognizing T or B cells that have escaped central tolerance, and it is mediated by inducing anergy, deletion by apoptosis, or regulatory cells of self-recognizing T and B cells. Genetic and epigenetic factors disturb immune tolerance (4). Loss of immune tolerance to self-antigens results in the development of autoimmunity and autoimmune diseases (5–8).

Though paradoxical, immunodeficiency and autoimmunity may occur simultaneously. Recent studies of two rare monogenic inborn errors of immunity (IEI) associated with immunodeficiency and autoimmunity—autoimmune polyendocrinopathy-candidiasis-ectodermal dystrophy (APECED) and immunodysregulation-polyendocrinopathy-enteropathy-X‐linked (IPEX)—have established the critical role of transcription regulators [autoimmune regulator (AIRE)] that regulate the transcription of numerous self-antigens in central tolerance and of Forkhead Box P3 (FoxP3), which is expressed in CD4 Treg, CD8 Treg, and T follicular regulatory cells (T_FR_), in suppressing autoreactive T cells in the periphery, which is called peripheral tolerance (9, 10). Recently, plasma cells with regulatory properties have been reported in experimental models of autoimmune and infectious diseases (11–13). Shen et al. (12) demonstrated that CD138^hi^ plasma cells produce both IL-35 and IL-10. IL-35 limits experimental autoimmune encephalitis *via* inhibition of pathogenic TH_1_ and TH_17_ cells, and in the *Samonella* infection model, IL-10 inhibits anti-*Salmonella* immunity. These regulatory plasma cells express surface IgM, CD80, CD86, CD40, CD69, CD44, TACI, CXCR4, MHC II, Tim1, and *Blimp1*. Lino et al., in a murine model of *Salmonella typhimurium* infection, reported that IL-10-producing CD138^+^ plasma cells express LAG-3, PD-L1, PD-L2, CD200, and BLIMP1 (13). The role of regulatory plasma cells in humans has not been explored. Furthermore, plasma cells are reduced in common variable immunodeficiency (CVID); therefore, it is unlikely that they play a significant role in the pathogenesis of CVID or autoimmunity and autoimmune diseases associated with CVID.

Autoimmunity and autoimmune diseases are observed with increased frequency in several IEI (14, 15). Autoimmunity and autoimmune diseases are common complications in CVID, affecting at least 25% of patients and may be the first presenting non-infectious manifestations (16–22). Both organ- and tissue-specific systemic autoimmune diseases are associated with CVID, with autoimmune cytopenia (e.g., immune thrombocytopenia, autoimmune hemolytic anemia) being the most frequent autoimmune manifestations. Several mechanisms have been reported to explain autoimmunity associated with CVID. These include increased T helper type 1 (T_H1_), T_FH_ cells, and CD21^low^ B cells and decreased CD4^+^CD25^+^FoxP3^+^ regulatory cells [reviewed in (23)].

Several investigators have studied CD4 Tregs in CVID (24–33); however, only limited data are available for other members of the regulatory lymphocyte club (33, 34). Here, we review them in-depth and their possible role in the pathogenesis of CVID and autoimmunity associated with CVID.

## Germinal Center Reaction and Its Regulation

The two important events in effective immune response, the class-switched recombination (CSR) and somatic hypermutation (SHM) or affinity maturation, resulting in generating high-affinity protective antibodies, occur in the dark zone of germinal centers (35, 36). However, clones of self-reactive B cells that are not eliminated can initiate autoantibody production in germinal centers (GCs) (35). There is evidence to support that self-reactive B cells are generated by SHM in GCs (35). The survival of these self-reactive B-cell clones depends upon the location and concentrations of autoantigens in GCs. SHM-mediated alteration of the antigen specificity of GC B cells can also play an important role in preventing autoantibody production in peripheral lymphoid tissues (36).

The regulation of the GC occurs at multiple levels and by multiple mechanisms. Several mechanisms have been proposed to explain B-cell autoimmunity, including chronic infection, molecular mimicry, excess production of memory B cells with a CD21^lo^ phenotype, IL-21 produced by T follicular helper cells (T_FH_), and regulatory lymphocyte dysfunctions. In this article, we also reviewed the role of CD4 Treg, CD8 Treg, and T_FR_ cells in GC reaction.

### TFH Cells and GC Reaction

CD4^+^ T cells that express high levels of the chemokine receptor CXCR5 migrate to GCs and regulate GC formation, selection of high-affinity antibody-producing B cells, isotype class switching, and generation of long-lived memory B cells and plasmablasts (37–40). In addition to CXCR5 expression, T_FH_ cells also express transcription factor B-cell lymphoma-6 (Bcl-6), programmed cell death receptor-1 (PD-1), inducible T-cell co-stimulator (ICOS), and CD40 ligand (CD40L/CD154) (41, 42). IL-21, the signature cytokine of T_FH_ cells, signaling the JAK and STAT pathway, supports the proliferation, survival and SHM, and differentiation of B cells to antibody-producing cells and long-lived memory B cells. Martin and colleagues (43), based upon the expression of CXCR3 and CCR6 markers, have divided T_FH_ cells into T_FH_1 (CXCR5^+^CXCR3^+^CCR6^−^), T_FH_2 (CXCR5^+^CXCR3^−^CCR6^−^), and T_FH_17 (CXCR5^+^CXCR3^−^CCR6^+^) cells. T_FH_2 and T_FH_17 cells are able to help naive B cells to differentiate to produce antibodies; however, all subsets of T_FH_ cells can induce differentiation of memory B cells to antibody-producing cells.

An increased T_FH_ cell response in the GC is associated with the expansion of low affinity and autoreactive B cells, and overactive T_FH_ cells are observed in a variety of systemic autoimmune diseases (44–48). Therefore, balanced responses of T_FH_ and B cells are required to eliminate pathogens and simultaneously prevent autoimmune disease.

### CD4 Treg Cells and T Follicular Regulatory Cells in GC Reaction

CD4 T cells with regulatory activity were originally described in 1982 by Damle and Gupta (49), who demonstrated that CD4^+^ T cells upon activation *in-vitro* suppressed proliferative responses of T cells to phytohemagglutinin and alloantigens in mixed lymphocyte culture reaction. In 1995, Sakaguchi and colleagues further defined CD25^+^ subsets of CD4 T cells with regulatory activity and termed them as Treg cells (50). In 2003, Tregs were further defined by the presence of transcription factor FoxP3 (51). The significance of the FoxP3 transcription factor in immune tolerance was reported in IPEX in which mutation of FoxP3 resulted in the development of autoimmunity.

The role of CD4 Tregs in the suppression of T cells and antibody responses is well established. Sakaguchi and colleagues (50) reported that depletion of CD4^+^CD25^+^ T cells leads to induction of antiparietal cell antibodies by gastric epithelia and of antithyroglobulin antibodies by thyroid follicular cells. Leonardo and colleagues (52) demonstrated the role of CD4 Tregs on germinal center formation and antibody response in a mouse model in which CD4 Tregs express the primate diphtheria toxin receptors. In these mice, depletion of specific CD4 Tregs resulted in enhanced GC formation, T_FR_ cell expansion, and autoantibody responses. Strongly enhanced GC/_TFH_ responses are also observed in patients with IPEX (53). Lim et al. (54, 55) reported that Foxp3^+^ Tregs can also directly suppress B-cell response without the need to first suppress T helper cells. Following activation, a subset of CD4 Tregs (CD4^+^CD25^+^CD69^−^) acquires CXCR5 expression while losing CCR7, allowing them to migrate to the B-cell follicle and suppress B-cell responses including B-cell survival, expression of activation-induced cytosine deaminase, and immunoglobulin production (56). Therefore, a subset of CD4 Tregs (CD4^+^CD25^+^CD69^–^) appears to transition to T_FR_ cells, and this subset of CD4 Tregs regulates antibody responses in GC by suppressing T_FH_ cells. T_FR_ cells were not normally defined until 2011, when three groups simultaneously defined T_FR_ cells as CXCR5^+^PD-1^+^BCL6^+^Foxp3^+^ cells (57–59). T_FR_ cells appear to have critical roles in controlling both foreign antigen-specific and self-reactive B cells. T_FR_ cell differentiation and maturation are facilitated by DCs and B cells (57). T_FR_ cells prevent T_FH_ cell-induced activation of autoreactive B cells. T_FR_ cells modify GC reaction by controlling the size of GCs and the selection of antigen-specific T_FH_ cells and B-cell clones and by regulating immunoglobulin isotype switch and affinity maturation of antibodies (60). The precise molecules that are responsible for such effects are unknown; cognitive interactions *via* CTLA-4 appear to mediate suppression (61, 62).

Fu and colleagues (63) studied the role of T_FR_ cells in autoimmunity in *Bcl6^fl/fl^Foxp3Cre* KO mice. These mice, as they age, develop spontaneous autoimmune diseases, associated with increased number of T_FH_ cells, production of autoantibodies, and IgG deposition in the kidney, supporting the role of T_FR_ cells in germinal center formation and control of autoimmunity. T_FR_ cells have been studied in a variety of autoimmune diseases (64–71). Increased T_FR_ cells are associated with decreased autoantibodies and stable disease in rheumatoid arthritis (66). An imbalance between T_FR_/T_FH_ cells correlates with disease activity in a number of autoimmune diseases (67–71).

### CD8 Treg Cells and GC Reaction

In addition to T_FR_ cells, CD8 Tregs also regulate GC reaction *via* regulation of T_FH_ and B-cell responses. CD8 Tregs (suppressor CD8) in humans were discovered in the early 1980s (49, 72). In the last 10 years, the role of CD8 Tregs in immune tolerance and experimental models and autoimmune diseases has been reported (73–77). The TCR repertoire differs between CD4 Tregs and CD8 Tregs: oligoclonal in CD8 Tregs (78) and diverse TCR repertoire in CD4 Tregs (79). In humans, FoxP3^+^CD8^+^ Treg cells are present in the thymus and tonsils and, in low frequency, in peripheral blood (80–83).

Several subsets of CD8^+^ Treg cells have been described in mice and humans [reviewed in (84–86)]. Shi et al. (87) reported that human central memory CD183^+^CD8^+^ T cells contain regulatory activity against T-cell responses mediated by IL-10. We further characterized CD8 Tregs both phenotypically and functionally (88–90). We examined the effect of CD183^+^CD45RA^−^CCR7^+^CD8^+^ T cells on various subpopulations of cells and observed that CD183^+^CD45RA^−^CCR7^+^CD8^+^ T cells suppress plasmablasts only. Furthermore, they did not have any significant effect on BAFF-R expression, suggesting that CD8 Tregs do not regulate B-cell survival (88). We further examined the direct effect of CD8 Tregs on B cells and demonstrated that CD8 Tregs as defined by CD183^+^CD25^high^CD278^+^CD8^+^ have greater inhibitory activity against B-cell proliferation and immunoglobulin production than CD183^+^CD45RA^−^CCR7^+^CD8^+^ Tregs (89). Kasahara and colleagues (90) demonstrated that CD8 Tregs (CD8^+^CD25^high^ICOS^+^CD183^+^) under T_FH_ differentiation conditions suppressed naive CD4 T-cell differentiation to T_FH_. We have also observed that CD8 Tregs regulate the induction of FoxP3 in CD4 T cells. CD8 Tregs appear to regulate GC reactions and GC development *via* their influence on both T_FH_ cells and directly on B cells and possibly *via* regulating CD4 Tregs. It is unclear if CD8 Tregs also regulate T_FR_ cells and Bregs.

### Breg Cells and GC Reaction

Regulatory B cells are immunosuppressive cells that downregulate immune responses and maintain immunological tolerance (91, 92). In 1974, Katz and colleagues (93) reported B cells suppressing the delayed type of hypersensitivity. However, it is in the last decade that Bregs have been investigated for their role in immune homeostasis and tolerance. Following exposure to the autoantigen, B cells mature into Breg cells that can express PD-1 and PD-L1, and suppress inflammation in autoimmune diseases *via* PD-1–PD-L1 interactions. In mice, B cells regulate immune responses through the release of IL-10, TGFβ, and IL-35 (91). In mice, IL-35 produced by plasma cells plays an important role in the negative regulation of immunity during autoimmune and infectious diseases (12). The role of IL-35 in B-cell-mediated negative regulation of immunity in humans has not been studied in detail. In a single report, Ye et al. (94) reported decreased plasma IL-35 and IL-35^+^ plasma cells in early-onset SLE patients. Bregs downregulate T- and B-cell immune responses *via* IL-10. In addition, Bregs promote the generation of CD4 Tregs and induce suppressive natural killer T cells [reviewed in (95)].

In humans, B cells regulate immune responses by secreting IL-10 and TGFβ (96, 97). Distinct subsets of B cells, namely, CD24^hi^CD38^hi^ (similar to transitional B cells) and CD24^hi^CD27^+^ (memory B cell, B10 cells), and CD25^+^CD71^+^CD73^−^ display regulatory activities. Although CD19^+^CD24^hi^CD38^hi^ Bregs are enriched in IL-10^+^ B-cell fraction in peripheral blood (95, 96), CD24^hi^CD27^+^ Bregs (B10) are relatively more suppressive for T-cell proliferation and IL-17/IFNγ expression. Both subsets produce IL-10; however, CD24^hi^CD27^+^ Bregs are enriched in TGFβ and granzyme B (96). Therefore, these two phenotypically distinct Bregs mediate immunosuppression *via* distinct mechanisms. Achour et al. (98) also reported that human Bregs inhibit T_FH_ cell differentiation and maturation and inhibit T_FH_-mediated antibody production *via* the expression of IL-10 and TGFβ and the expansion of CD4 Tregs. In their study, Bregs were CD19^+^CD24^hi^CD38^hi^CD25^+^CD71^+/−^CD73^−^PD-L1^+^ICAM1^+^ICOS-L^+^IL-21R^−^CD80^hi^CD86^hi^. Therefore, Breg cells regulate GC reaction *via* suppressing T_FH_ cells and promoting the generation of CD4 Treg cells.

The role of Breg cells has been reported in a variety of autoimmune diseases (91, 92, 95, 99–101). Unlike other regulatory lymphocytes that express FoxP3, Bregs do not express FoxP3, and a specific transcriptional factor of Breg cells has not been discovered.

## Germinal Center Reaction in CVID

CVID is characterized by severely reduced numbers of circulating class-switched memory B cells and reduced levels of SHM resulting in impaired pathogen-protective high-affinity antibody response (102–108). Therefore, GCs as the primary site for both CSR and SHM may be disturbed in CVID patients.

Unger and colleagues (109) studied lymph node biopsies from CVID patients with lymphadenopathy. In the majority of cases, varying degrees of ill-defined GC hyperplasia were observed that correlated with the increased percentage of circulating CD21^low^ B cells. Class-switched plasma cells were severely reduced. Therefore, large GCs and the reduction of circulating memory B cells and class-switched plasma cells suggest a failure of GC output rather than GC formation in CVID patients with lymphadenopathy.

van Schouwenburg and colleagues (110) studied naive and the antigen-selected BCR repertoire in CVID patients and were able to identify the GC reaction as the process most often deregulated in CVID patients. They also observed that some patients have possible defects in early B-cell development or selection against autoimmune features. Their study indicated that in the majority of CVID patients, repertoire formation is intact, while repertoire specification is often impaired. Therefore, CVID patients, in addition to having a quantitative defect in B-cell development, also had impaired quality of B cells.

### T_FH_ Cells in CVID

As discussed above, T_FH_ cells play an important role in GC reaction. CVID patients with ICOS deficiency show severely impaired GC formation in lymphoid tissues and severely decreased blood memory T_FH_ cells, accompanied by a severe deficiency of memory B cells. Bossaller et al. (111) and Grimbacher et al. (112) reported decreased proportions of CXCR5^+^CD4^+^ T_FH_ cells in CVID patients with ICOS deficiency. Cunill et al. (113) analyzed T_FH_ cells in CVID patients. Patients were divided into smB^−^ (<2% switched memory B cells) and smB^+^ (switched B cells >2%). They observed an increased percentage of CD4^+^CXCR5^+^ T_FH_ cells in CVID as compared with controls; however, these differences were observed only between smB^−^ CVID patients. These T_FH_ cells have increased PD-1 expression. Coraglia et al. (114) studied T_FH_ cells in 21 CVID patients divided into group I with autoimmune/granulomatous (AI/GD) diseases and group II without AI/GD. They observed increased CD4^+^CXCR5^+^ T_FH_ cells in group I as compared with group II and healthy controls. Group II was not different from healthy controls. When data were analyzed for CCR7 and PD-1 expression, CD4^+^CXCR5^+^CCR7^lo^PD-1^hi^ cells were universally present in group I but not in group II. Kasahara et al. (115) reported decreased T_FH_ cells expressing PD-1 and ICOS-1 and reduced IL-21 secretion but a normal function of T_FH_ cells in CVID patients suggesting intrinsic B-cell defect. Borte et al. (116) reported that exogenous IL-21 restored immunoglobulin production in CVID. They reported decreased IL-21 mRNA in T cells; however, they did not find any mutation in IL-21. They did not examine IL-21 secretion.

Several investigators have reported increased levels of the T_FH_1 subset in CVID patients with splenomegaly and/or AI/GD, when compared with CVID patients without AI/GD or splenomegaly and healthy controls. Cunill et al. (113), Unger et al. (117), and Kasahara et al. (115) have observed increased cT_FH_1 cells in CVID. Cunill et al. (113) observed a significant increase in T_FH_1 cells in smB^−^ CVID patients. Unger et al. (117) observed increased T_FH_1 in patients with autoimmune manifestations, and the strongest shift in T_FH_1 cells was observed in CVID with increased CD21^low^ B cells.

Cuhill et al. (113) and Kasahara et al. (115) did not observe any significant difference in T_FH_2 cells between CVID and controls. Several investigators reported decreased T_FH_17 cells in CVID patients (113, 115–118). Reduced production of IL-17 by CD4^+^ T cells has been associated with the reduced number of CD27^+^IgD^−^ B cells in CVID patients and healthy subjects (119, 120). Cunill et al. (113) observed reduced T_FH_17 cells and increased T_FH_1/T_FH_17 ratio in smB^−^ CVID patients. Berrón-Ruiz and colleagues (119) observed decreased IL-17A production in CVID. Barbosa et al. (120) also observed decreased IL-17 in CVID that correlated with increased CD21^low^; however, they did not observe any correlation in CVID with autoimmunity and autoimmune disease.

### T_FR_ Cells in CVID

T_FR_ cells appear to have critical roles in controlling both foreign antigen-specific and autoreactive B cells. T_FR_ cells suppress antibody responses by suppressing T_FH_ cells (57–61). Fu et al. (6) reported an association between T_FR_ cell deficiency and the development of autoimmunity in mice. Cunill et al. (113) reported a reduction in CXCR5^+^CD25^hi^CD127^low^ T_FR_ cells in CVID patients as compared with controls. Furthermore, a significant reduction was observed in smB^−^ CVID but not in smB^+^ CVID patients. They demonstrated that the sorted CXCR5^+^CD25^hi^CD127^low^ T_FR_ cells from smB-1 CVID had decreased regulatory activity. Kasahara et al. (115) investigated T_FR_ cells (CD4^+^CD45RA^−^CXCR5^+^CD25^+^FoxP3^+^) in CVID patients with (*n* = 12) and without (*n* = 20) autoimmune diseases. They observed a decreased percentage of T_FR_ cells in CVID patients; however, no significant difference was observed between the autoimmunity and non-autoimmunity groups. They also observed an increase in the T_FH_/T_FR_ ratio in CVID patients with autoimmune diseases as compared with controls but not between CVID patients without autoimmune diseases and controls. Coraglia et al. (114) reported similar proportions of T_FR_ cells (CD4^+^CXCR5^+^FoxP3^+^) in CVID patients and healthy controls. Furthermore, no difference in T_FR_ cells was observed between CVID patients with or without AI/GD.

### CD4 Treg Cells in CVID

A number of investigators have reported decreased CD4 Tregs in freshly isolated mononuclear cells (nTregs) in patients with CVID (24, 26–34, 119, 120). However, Kutukculer et al. (25) in 20 pediatric CVID patients reported no change in CD4 Tregs regardless of the severity of disease, and the presence of autoimmunity was not associated with decreased CD4 Tregs. Melo et al. (30), using the CD4^+^CD25^high^127^low^FoxP3^+^ phenotype as criteria for CD4 Tregs, reported decreased CD4 Tregs in CVID; however, they observed no difference between those with and without autoimmunity. Romberg et al. (34) also reported reduced frequency of CD4 Tregs (CD4^+^CD25^hi^CD127^lo^) in CVID patients especially CVID with autoimmune cytopenia. Furthermore, they reported that CD4 Tregs from CVID with autoimmune cytopenia were impaired in suppressing allogeneic T cells of healthy controls. There was an inverse relationship between the expansion of T_FH_ and CD4 Tregs in CVID with autoimmune cytopenia. Cunill et al. (113) examined CD4 Tregs (CD4^+^CD25^high^CD127^low^) in 22 CVID patients (*n* = 22) and observed decreased proportion in CVID as compared with healthy controls. Furthermore, reduced CD4 Tregs were observed in smB^−^ CVID patients but not in smB^+^ CVID patients. They demonstrated that CD4^+^ Tregs had regulatory activity; however, they did not compare the functions of CD4 Tregs between controls and CVID patients. Furthermore, they did not analyze their data in relation to autoimmunity. Louis et al. (24) reported decreased CD4 Tregs in CVID patients with mutation of the inositol trisphosphate kinase beta (*ITPkβ*) gene. Horn et al. (31), using two different phenotypic markers (CD4^+^CD25^high^CD127^low^FoxP3 and CD4^+^CD25^high^FoxP3^+^CTLA-4^+^), reported decreased CD4 Treg cells in CVID patients with granulomatous disease and immune cytopenia. Several other investigators have reported an association between reduced CD4 Tregs and CVID with autoimmune diseases (29, 31, 32). Kofod-Olsen et al. (32) reported an association between decreased CD4^+^ Tregs and increased pro-B10 Breg cells and autoimmune phenomenon in CVID. Carter et al. (28), using the CD4^+^CD25^+^FoxP3^+^CTLA-4^+^ phenotype, reported decreased CD4 Treg cells in a small cohort of CVID patients with autoimmunity. In contrast, Arumugakani et al. (29) observed decreased proportions and numbers of CD4 Tregs in a CVID group with splenomegaly; however, they observed comparable proportions and numbers in CVID with or without autoimmunity. Fevang et al. (121) also reported decreased CD4 Tregs in CVID patients with splenomegaly as compared with those without splenomegaly; however, they did not observe a significant difference in CD4 Tregs between CVID patients with idiopathic thrombocytopenia or granuloma. Yu et al. (122) examined both the numbers (CD4^+^CD25^high^CD127^lo^) and functions of CD4 Tregs in 14 CVID patients (8 with and 6 without autoimmunity) and 5 healthy controls. Patients with CVID both had reduced numbers and function of CD4 Tregs. Furthermore, the degree of CD4 Treg cell dysfunction correlated with the expression of FoxP3, granzyme A, and pSTAT3. Klemann and colleagues (123) reported decreased CD4 Tregs in CVID patients with pathogenic mutation of *NF-κB2*; however, there was no correlation with autoimmunity. In none of the published reports have investigators examined *ex-vivo* activated CD4^+^ Tregs (iCD4 Tregs). Yesillik et al. (33), using the CD4^+^CD25^high^127^low^FoxP3^+^ phenotype to define CD4^+^ Tregs, observed decreased proportion of both nCD4^+^ Tregs and iCD4^+^ Tregs (*ex-vivo* activation of T cells with anti-CD3/CD28) in CVID; however, they did not observe any significant difference between the autoimmune and non-autoimmune disease groups. This could be due in part to the small sample size of patients with autoimmunity.

### CD8 Treg Cells in CVID

CD8 Tregs suppress the differentiation of T_FH_ cells from naive CD4 T cells and are shown to regulate both T- and B-cell responses and GC reaction. CD8 Tregs regulate immune response and development of several experimental models of autoimmune diseases and in human autoimmune diseases (77, 124–126); however, CD8 Tregs have not been studied in detail in patients with IEI. In humans, Shi and colleagues (87) have shown that CD8 Tregs (CD8^+^CD183^+^CXCR3^+^) regulate T-cell proliferation and effector functions. CD8 Tregs as defined by CD8^+^CD25^high^FoxP3^+^ play an important role in the maintenance of self-tolerance (125). Churlaud et al. (83), Wang et al. (127), and Lu et al. (124) have demonstrated that CD8^+^CD25^+^FOXP3^+^ Treg cells that have been expanded *in vitro* inhibit the proliferation of CD4^+^ and CD8^+^ T cells. We have reported alterations in iCD8 Tregs in patients with selective IgM deficiency (128), Good syndrome (129), syndrome of selective IgM deficiency, severe T-cell deficiency and *Mycobacterium avium complex* infection (130), and hypogammaglobulinemia associated with CMV colitis and deficiency of CMV-specific CD8 T cells (131). Yesillik et al. (33) recently analyzed both nCD8 Tregs (without *in-vitro* activation) and iCD8 Tregs (*ex vivo* activated with anti-CD3/CD28) in 25 subjects with CVID. The proportions of iCD8 Tregs were significantly reduced in CVID; however, no significant difference was observed in nCD8 Tregs between CVID and controls. Furthermore, they did not observe any difference in the proportions of iCD8 Tregs between CVID with or without autoimmunity. Therefore, CD8 Tregs need to be studied both phenotypically and functionally in a large cohort of CVID patients with and without autoimmunity to delineate their role in autoimmunity-associated CVID.

### Breg Cells in CVID

Breg cells regulate T- and B-cell responses including the maintenance of CD4 Tregs (88, 89, 95). B regulatory cell frequency and functions are decreased in a number of systemic autoimmune diseases (92, 93, 95, 96). Barsotti et al. (132) studied two subsets (CD19^+^CD24^hi^CD38^hi^ and CD19^+^CD24^hi^CD27^+^) of Bregs in 42 patients with CVID and healthy controls. Both these populations with or without activation with CpG or LPS to express IL-10 were significantly decreased in CVID as compared with controls. Furthermore, IL-10 production by sorted B cells was significantly lower in CVID as compared with controls. No significant difference was observed in CVID patients with or without autoimmune diseases. However, when data were analyzed between CVID patients with autoimmune cytopenia and gastrointestinal autoimmune disease, the frequency of CD24^hi^CD38^hi^ was significantly decreased in the cytopenia group as compared with the gastrointestinal autoimmunity group and healthy controls. Therefore, Bregs may have a differential effect on autoimmune manifestations associated with CVID. Furthermore, they did not observe any correlation between the frequency of Breg and CD4 Treg cells. We also observed decreased proportions of CD19^+^CD24^hi^CD38^hi^ regulatory B cells in CVID patients as compared with controls; however, we did not observe any correlation with autoimmunity in CVID (30). Vlkova et al. (133) investigated Breg cells by stimulating peripheral blood B cells *via* stimulation of T cells by a plastic-coated anti-CD3 monoclonal antibody for 72 h and adding PMA and ionomycin for the last 4 h. They observed no difference in the frequency of CD19^+^CD24^hi^CD38^hi^ Breg cells. However, they did observe a significantly reduced frequency of CD19^+^CD24^hi^CD38^hi^IL-10^+^ Bregs in CVID as compared with controls. No relationship was observed with the EUROclass categories of CVID. Furthermore, they observed an impaired Breg function in CVID as demonstrated by failure to suppress IFNγ and TNFα production by CD4^+^ T cells and an increased number of CD4^+^IFNγ^+^TNFα^+^ cells. In contrast, Arumugakani et al. (29) used CpG + rhCD40L to stimulate B cells for 43 h and analyzed Breg cells, which they termed pro-B10 cells, as CD19^+^IL-10^+^. They observed an increased frequency of pro-B10 Breg cells in total CVID patients as compared with controls. Furthermore, they observed an even more significant increase in pro-B10 cells in the CVID with autoimmunity group as compared with controls, whereas the frequency of pro-B10 cells in the non-autoimmune group was similar in total controls. In addition, they observed a correlation with EUROclass categories. They also did not observe any correlation with decreased CD4 Tregs. Different experimental conditions and different phenotypic criteria to define Breg cells may account for the discrepancies among these studies. An increase in Breg cells has also been reported in patients with primary selective IgM deficiency (126).

## Summary and Concluding Remarks

B-cell clones expressing self-reacting BCRs in GCs can initiate autoantibody production. Peripheral tolerance is induced by CD4 Treg, CD8 Treg, T_FR_, and Breg cells that regulate GC reaction by multiple mechanisms, including anergy, apoptosis, and suppression of effector functions of self-reacting T and B cells. Furthermore, these regulatory lymphocytes regulate themselves (regulators of regulatory lymphocytes). In the majority of CVID studies, regulatory lymphocytes have been phenotypically examined, and their functions have been examined in very few studies. There is a general consensus with regard to decreased CD4 Tregs in CVID; however, there are conflicting data regarding their relationship with autoimmunity. A subset of CD4 Tregs (CD4^+^CD25^+^CD69^−^), migrating to lymphoid organs and transitioning into T_FR_ cells, suppresses antibody response. Data on T_FR_ cells are conflicting. CD8 Tregs regulate directly both T_FH_ and B-cell responses. There are very little data about CVID. Similarly, Breg cells have not been studied in detail. The role of regulatory lymphocytes in the pathogenesis of low immunoglobulins in CVID remains to be explored. Since regulatory lymphocytes regulate each other, this poses another challenge to sort out the role of individual regulatory lymphocytes in the pathogenesis of CVID. There appears to be a phenotypic heterogeneity in subsets of CD8 Treg, Breg, and T_FR_ cells. Perhaps a multicenter comprehensive study of both the phenotypic and functional analyses of regulatory lymphocytes in a well-categorized large cohort of CVID patients is needed to delineate their role in the pathogenesis of CVID and associated autoimmunity and autoimmune diseases. Furthermore, additional studies are needed to examine the effect of biologics on regulatory lymphocytes in CVID patients with autoimmune diseases.

## Author Contributions

SG conceptualized, formatted, wrote, and edited the manuscript. YD and AG wrote part of the paper and edited the manuscript. All authors contributed to the article and approved the submitted version.

## Conflict of Interest

The authors declare that the research was conducted in the absence of any commercial or financial relationships that could be construed as a potential conflict of interest.

## Publisher’s Note

All claims expressed in this article are solely those of the authors and do not necessarily represent those of their affiliated organizations, or those of the publisher, the editors and the reviewers. Any product that may be evaluated in this article, or claim that may be made by its manufacturer, is not guaranteed or endorsed by the publisher.
